# CypB promotes cell proliferation and metastasis in endometrial carcinoma

**DOI:** 10.1186/s12885-021-08374-7

**Published:** 2021-06-29

**Authors:** Jing Liu, Ying Zuo, Gui-Mei Qu, Xiao Song, Zhong-Hui Liu, Ting-Guo Zhang, Zhu-Hua Zheng, Hong-Kun Wang

**Affiliations:** 1 Department of Pathology, Affiliated Yantai Yuhuangding Hospital, Medical College of Qingdao University, Yantai, China; 2 Department of Gynecology, Affiliated Yantai Yuhuangding Hospital, Medical College of Qingdao University, Yantai, China; 3 Department of Pathology, People’s Hospital of Rong cheng, Weihai, China; 4 Department of Pathology, Yantai Muping District Traditional Chinese Medicine Hospital, Yantai, China; 5 Department of Pathology, School of Medicine, Shandong University, Jinan, Shandong China; 6 Department of Pediatrics, Traditional Chinese Medicine Hospital of Rushan, Weihai, China; 7 Department of Gynaecology and Obstetrics, Shanghai General Hospital, Shanghai Jiao Tong University School of Medicine, Shanghai, China

**Keywords:** Endometrial cancer, Cyclophilin B, Proliferation, Microarray

## Abstract

**Background:**

The molecular pathogenesis of endometrial cancer is not completely understood. CypB upregulated in many cancers, however, its role in endometrial carcinoma has not been studied. Here, we determine the effect of CypB on the growth of endometrial cancer.

**Methods:**

In this study, we examined the expression of CypB in endometrial cancer tissues using immunohistochemistry. CypB silenced in HEC-1-B cell line by shRNA. CCK-8, colony formation assays, wound healing assays, and transwell analysis were performed to assess its effect on tumor cell proliferation and metastasis. Furthermore, microarray analysis was carried out to compare the global mRNA expression profile between the HEC-1-B and CypB-silenced HEC-1-B cells. Gene ontology and KEGG pathway enrichment analysis were performed to determine the potential function of differentially expressed genes related to CypB.

**Results:**

We found that CypB was upregulated in endometrial cancer, inhibit CypB expression could significantly suppress cell proliferation, metastasis, and migration. We identified 1536 differentially expressed genes related to CypB (onefold change, *p* < 0.05), among which 652 genes were upregulated and 884 genes were downregulated. The genes with significant difference in top were mainly enriched in the cell cycle, glycosphingolipid biosynthesis, adherens junctions, and metabolism pathways.

**Conclusion:**

The results of our study suggest that CypB may serve as a novel regulator of endometrial cell proliferation and metastasis, thus representing a novel target for gene-targeted endometrial therapy.

**Trial registration:**

YLYLLS [2018] 008. Registered 27 November 2017.

**Supplementary Information:**

The online version contains supplementary material available at 10.1186/s12885-021-08374-7.

## Background

Endometrial cancer represents a group of epithelial malignant tumors occurring in the endometrium and is one of the three most common malignant tumors of the female genital tract. Seventy-five percent of the patients are diagnosed in the early phase, and the 5-year survival rate was 65–92% [1, 2]. The molecular pathogenesis of endometrial cancer involves abnormalities in many genes and signaling pathways. For example, mutations in *P53*, increased microsatellite instability, mutations in *PTEN*, and abnormalities in the Notch signaling pathway all lead to uncontrolled cell proliferation and apoptosis, which in turn lead to the occurrence and development of endometrial cancer [3–6]. At present, the molecular pathogenesis of endometrial cancer is not entirely understood, and further research is warranted to develop successful therapies.

Cyclophilins are highly conserved proteins that are ubiquitously expressed intracellularly. They were first recognized as host cell receptors for the potent immunosuppressive drug cyclosporin A [7, 8]. They act as molecular chaperones that fold, translocate, and process newly synthesized proteins. There are 16 types of human cyclophilins, cyclophilins A (CypA) and CypB being the two most abundant and the most studied ones. Li et.al using proteomics first report cyclophilin A upregulated in endometrial carcinoma serve as a potential prognostic factor [9], and up-regulation of cyclophilin A could render resistance to chemotherapeutic-induced apoptosis in cancer cells [10]. CypA also been found to be up-regulated in paclitaxel-resistant endometrial cells, and knockdown of CypA could reverse the paclitaxel-resistant through suppression of MAPK kinase pathways [11].

Cyclophilin B (CypB) is a 21 kDa peptidyl-prolyl *cis*-*trans* isomerase [12] that is expressed in the endoplasmic reticulum (ER) lumen [13] and nucleus [14]. It has been implicated in hepatitis virus replication [15], immunosuppression [16], chemotaxis [17], and prolactin signaling [18]. Moreover, its increased expression may significantly contribute to the pathogenesis of human breast cancer [19], myeloma [20], hepatic carcinoma [21], gastric cancer [22], head and neck squamous cell carcinoma [23], and glioblastoma [24]. Finally, it has also been used as a serum biomarker for the early detection of pancreatic cancer [25]. However, to date, few study has investigated the role of CypB in endometrial cancer.

Hence, to determine the effect of CypB on the growth of endometrial cancer, we aimed to assess its expression in endometrial tissues and CypB-silenced HEC-1-B cells and measure the relative gene expression with microarray analysis.

## Methods

### Cell lines and transient transfection

The HEC-1-B cells were purchased from the Chinese Academy of Sciences (Shanghai, China). The cells were cultured in modified Eagle’s medium (Gibco, Life Technologies, Carlsbad, CA, USA) supplemented with 10% fetal bovine serum (FBS, Gibco, Grand Island, NY, USA). And cell lines were maintained at 37 °C in a humidified atmosphere of 5% CO_2_.

The lentivirus-expressed CypB-specific short hairpin RNA (shRNA) was used to knock down the expression of CypB and negative control shRNA (NC-shRNA) as control. These shRNAs were used for transfection in the HEC-1-B cell line following the manufacturer’s protocol.

### Sample collection

All the tissue samples were collected via biopsy of surgical resection without chemotherapy or radiotherapy between December 2017 and September 2018 in the department of Gynecology, Affiliated Yantai Yuhuangding Hospital (Yantai, Shandong, China). The study was approved by the Ethics Committee of Yantai Yuhuangding Hospital on November 27, 2017 (registration number: YLYLLS [2018] 008). All samples were collected after obtaining written informed consent. The samples were snap-frozen in liquid nitrogen and stored at − 80 °C before RNA extraction or generation of formalin-fixed, paraffin-embedded tissue sections for immunohistochemistry.

### Cell proliferation and clone formation

Cell proliferation was determined using the Cell Counting Kit-8 assay (CCK-8; Beyotime Biotechnology, Shanghai, China) per the manufacturer’s instructions. Cells in the logarithmic growth phase (1 × 10^4^ cells/mL per well) were grown in 96-well plates in medium containing 10% FBS in an incubator with 5% CO_2_ at 37 °C for 72 h after transfection. Afterward, 10 mL of CCK-8 solution was added to each well, and the plates were incubated for an additional 4 h. The absorbance in each well was measured at a wavelength of 450 nm with a microplate reader.

For clone formation, HEC-1-B cells were transfected with CypB-shRNA for 48 h and were collected and seeded in triplicate into 6-well plates at a density of 1000 cells/mL per well. The cells were incubated for 10 days at 37 °C in a 5% CO_2_ atmosphere. They were then fixed with 4% paraformaldehyde for 30 min and stained with Giemsa (Beyotime Biotechnology) for 20 min. After washing with double-distilled H_2_O several times, images of the cell plates were taken (Canon, Inc., Tokyo, Japan).

### In vitro wound healing

The wound-healing assay was performed to evaluate cell migration. Cells were seeded onto 35 mm dishes. After cells reached over 90% confluence, using a sterile pipette tip to make a scratch through the confluent monolayer. The medium was changed and cells were cultured for 24 h. The percent wound closure was calculated for four randomly chosen fields.

### Invasion assays

For the invasion assay, 105 cells in serum-free medium were placed into the upper chamber of the insert with Matrigel (BD Biosciences, Franklin Lakes). After 24 h of incubation at 37 °C, we removed the cells remaining in the upper chamber or on the upper membrane. The number of cells adhering to the lower membrane of the chambers was counted after staining with a solution containing 0.1% crystal violet (Beyotime Institute of Biotechnology, Beijing, China) and 20% methanol.

## RNA extraction, reverse transcription, qRT-PCR, western blot, and microarray analysis

Total RNA was extracted using TRIzol, and cDNA was synthesized with the PrimeScript RT reagent Kit (TaKaRa, Dalian, China). Gene expression was assessed by qRT-PCR using SYBR Premix Dimer Eraser (Perfect Real Time, TaKaRa) assay kits. Relative fold changes in expression were calculated using the comparative Ct (2^-∆∆Ct^) method.

Total protein was collected from cells treated with RIPA lysis buffer, separated by sodium dodecyl sulfate-polyacrylamide gel electrophoresis (SDS-PAGE) and then transferred onto PVDF membrane (Millipore, Bedford, MA). Primary antibodies used in this study were shown as follows: rabbit polyclonal antibodies for CypB, Ang2 (immunoway, USA), and VEGF (Weiao, China). Beta-actin protein (Santa Cruz, CA) was used as a loading control.

The RNA samples collected 72 h after lentivirus transfection were submitted to Phalanx Biotech (Hsinchu, Taiwan) for microarray analysis. We used the Phalanx Human OneArray Plus Gene Expression Profiling platform 6.1 to analyze the CypB-mediated alterations of mRNA expression.

### Immunohistochemistry (IHC)

Sections (4 μm) were cut from the constructed TMA blocks, deparaffinized, and rehydrated. Heat-induced epitope retrieval was performed onboard of the Leica Bond RX platform at 100 °C using EDTA buffer (pH 9.0, Leica) for 20 min, followed by 15 min of incubation with anti-CYPB antibody (#43603, Cell Signaling Technology, Danvers, MA, USA) or anti-β-catenin antibody (#8480, Cell Signaling Technology) at room temperature and with Bond™ Polymer Refine Detection kit (Leica Biosystems, Buffalo Grove, IL, USA) for 8 min. The reaction was visualized using 3,3′-diaminobenzidine tetrahydrochloride for 10 min and with hematoxylin as a counterstain. Scoring was performed by pathologists (MK, PR) using a Nikon Eclipse microscope on TMA glass slides at 20× magnification. Tissues were scored for CypB expression, and the scoring system reflected the extent and intensity of staining: the intensity was assigned a score of 0, 1, 2, or 3, representing negative, weak, moderate, or strong expression, respectively; while the extent was assigned a score of 0, 1, 2, 3 or 4, representing < 5, 6–25, 26–50, 51–75 and > 75% of cells stained. The overall quantitation of the score was obtained by multiplying the average intensity and score of five different high-power fields (at 400× magnification). The samples were divided into two groups based on final staining scores, which ranged from 0 to 7: the high expression group (scores of ≥4) and the low expression group (scores of < 4) [26].

### Gene ontology functional and pathway enrichment analysis

GO (gene ontology) and KEGG pathway enrichment analysis was used for differentially expressed genes (DEGs) using the DAVID database. FDR values of < 0.05 were set as the cut-off criterion for the two analyses.

### Statistical analysis

Statistical analysis was performed using SPSS software, version 18.0 (SPSS, Chicago, IL, USA). The chi-square test was used to determine the differences in age and tumor grades between high and low expressed CypB groups. Differences between two groups were analyzed using Student’s *t*-test for comparison of two groups or by one-way analysis of variance for comparison of more than two groups. *P* values of < 0.05 were considered statistically significant.

## Results

### CYPB was overexpressed in endometrial cancer

**Fig. 1:**
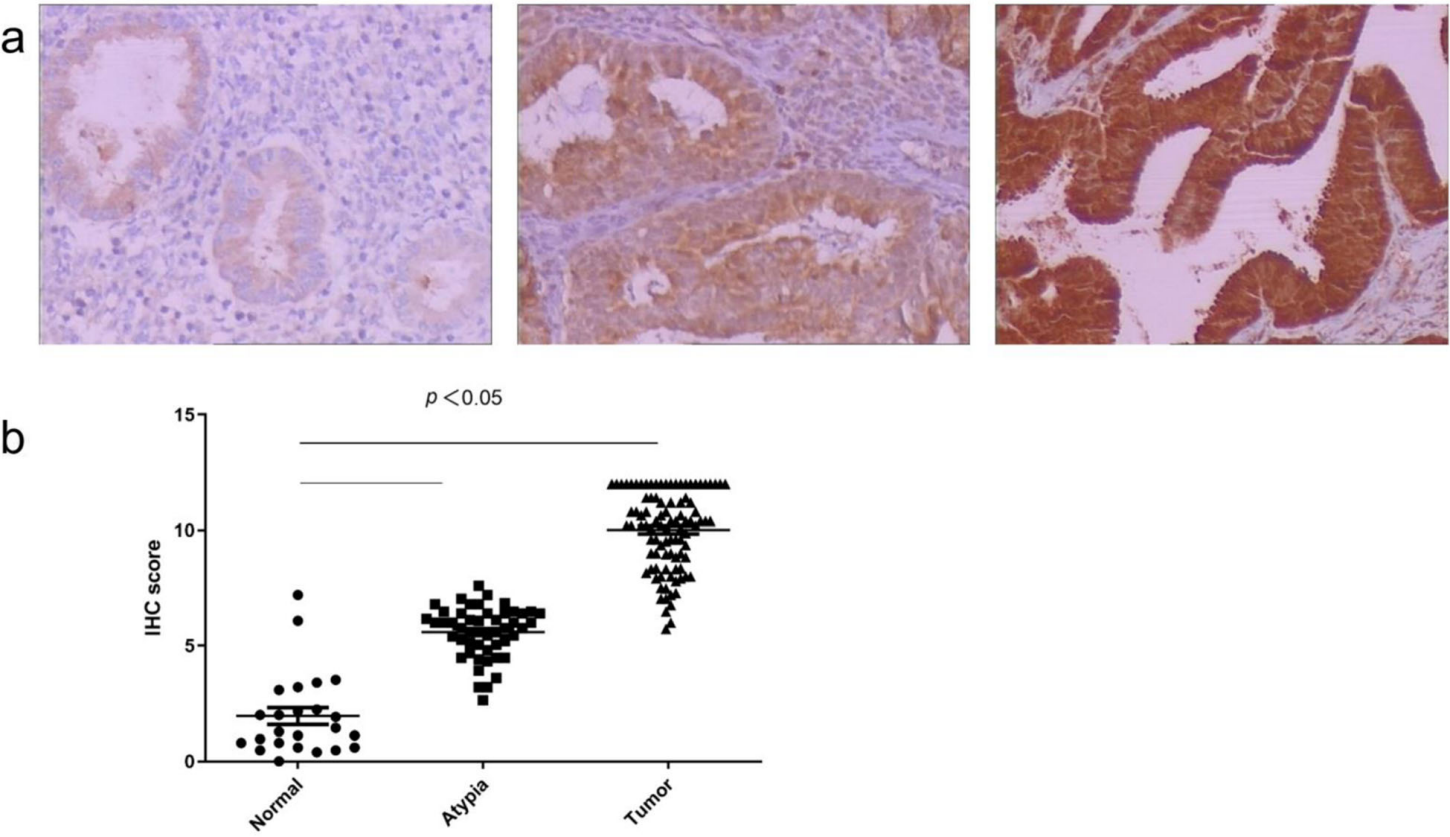
CypB is overexpressed in endometrial cancer. **a** Representative photomicrographs of immunohistochemical staining for CypB among endometrial tissue samples are presented. **b** Statistical analysis of relative CypB expression levels in normal tissues, atypic tissues, and endometrial tissues. These findings indicate that CypB was significantly upregulated in tumor tissues

Twenty-four normal endometrium tissue (control), 50 of atypical complex hyperplasia, and 96 endometrial cancer tissues were used to validate protein expression by immunohistochemistry. CypB expression was significantly higher in atypical complex hyperplasia and endometrial cancer tissues compared with normal endometrium tissues (Fig. 1), suggesting that higher expression of CypB is associated with the progression of endometrial cancer. Furthermore, no significant association was observed between CypB expression and patient age or tumor stages (Table 1).

**Table 1:** The CypB expression between age and tumor stages

		CypB expression levels			
		High	Low	Ratio (high/low)	P*
Characteristic	N(%)				
Ages					
<55	39	16	23		0.916
≥ 56	57	24	33		
Stage					
Tis + I	68	40	28	1.43	0.864
II + III	28	17	11	1.55	
Low (I + II)	85	50	35	1.43	
High (III)	8	7	1	7	

* Chi-square test was used

### Downregulation of CypB inhibits HEC-1-B cell proliferation and metastasis

**Fig. 2:**
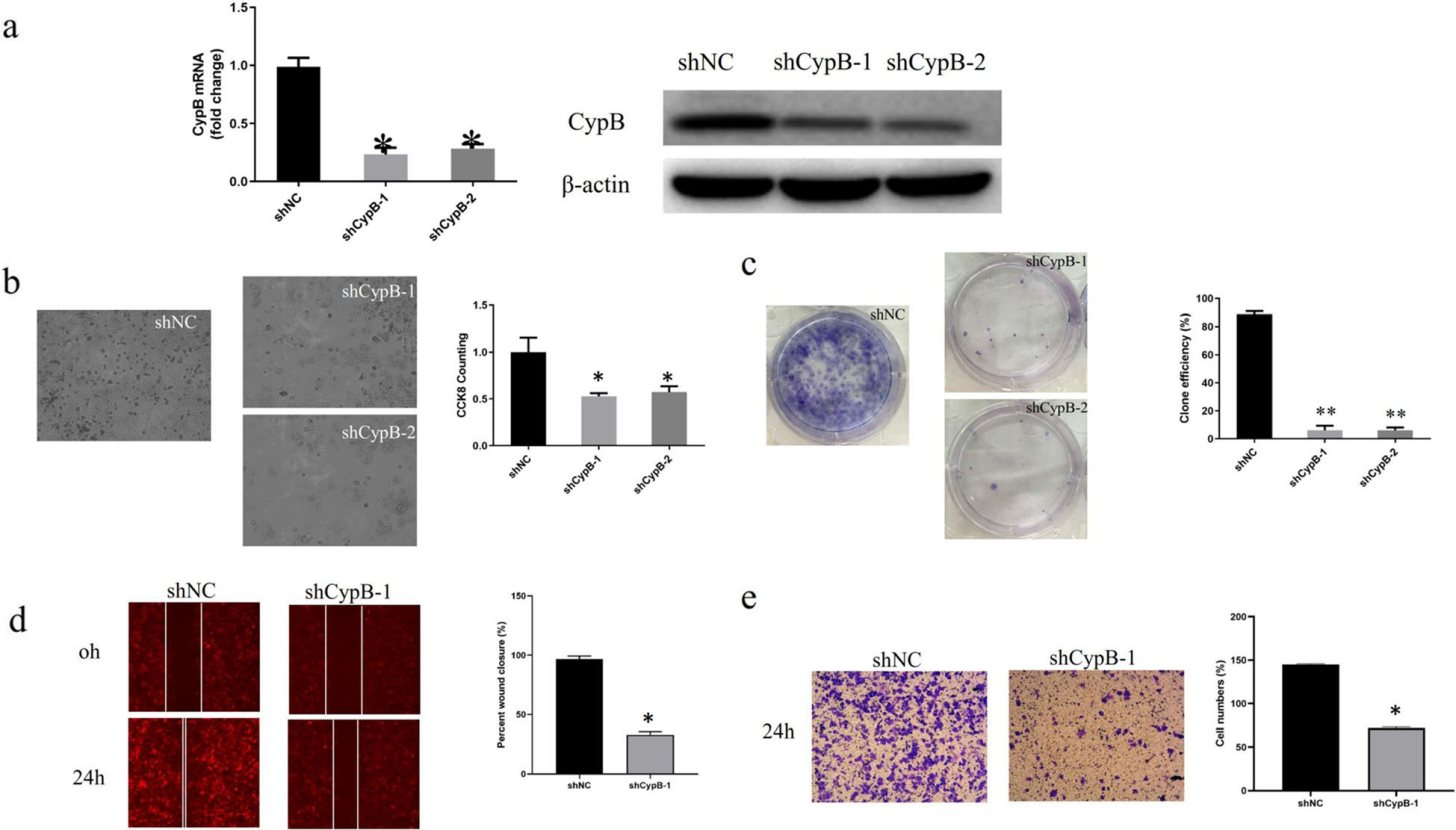
shRNA inhibited CypB expression efficiently in HEC-1-B cells, and CypB knockdown suppressed cell proliferation, colony formation, migration, and invasion in HEC-1-B cells. **a** shCYPB-1 and shCYPB-2 significantly downregulated the expression of CypB both in mRNA and protein level. The conventional microscope pictures of HEC-1-B cells treated with shNC or shRNAs (− 1 and − 2), and CCK-8 assay **b**, colony formation (**c**) assay, wound healing **d**, and transwell assays with matrigel (**e**) of HEC-1-B cells with CypB downregulation. This shows that CypB silencing significantly inhibited HEC-1-B cell proliferation and metastasis. (*p < 0.05, **p < 0.005)

To investigate the role of CypB in endometrial cancer, we treated the HEC-1-B cell line with CypB-shRNA. CypB-shRNA significantly reduced CypB expression both in mRNA and protein levels (Fig. 2a), indicating that a highly efficient knockdown of CypB expression was achieved.

Microscopic observation of the HEC-1-B cells was transfected with CypB-shRNA or NC-shRNA showed a decrease in cell proliferation after the downregulation of CypB. The results of the CCK-8 assay indicated that, compared to control cells, the proliferation rate of CypB-knockdown HEC-1-B cells decreased 72 h after transfection (Fig. 2b). *CYPB* silencing in HEC-1-B cells substantially reduced colony formation (Fig. 2c). The wound-healing assay showed that the downregulation of CypB in HEC-1-B cells was associated with significantly slow cell migration (Fig. 2d). Transwell assays with matrigel demonstrated that HEC-1-B CypB^-shRNA^ cells had a lower invasive activity than HEC-1-B ^*NC*-shRNA^ vector cells (72 ± 1 vs. 145 ± 1, *p* < 0.01; Fig. 2e). All these results demonstrated that downregulated CypB inhibits HEC-1-B cell proliferation and metastasis in vitro.

### Identification of DEGs in endometrial cancer cell with downregulated CypB expression

**Fig. 3:**
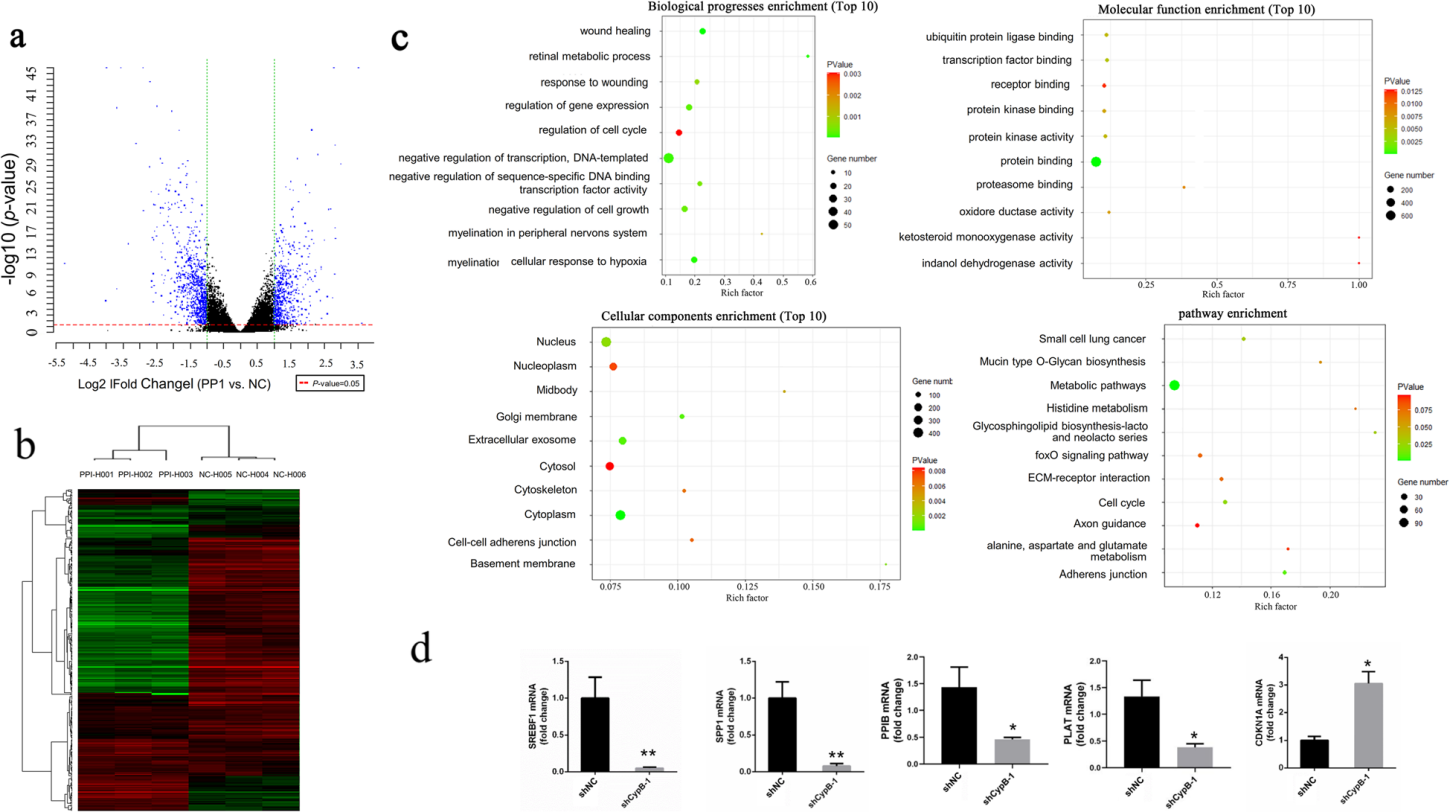
Identification of DEGs, GO functional annotation, and pathway enrichment analysis of the identified differentially expressed genes in CypB-downregulated and normal control HEC-1-B cells. **a** The vertical lines correspond to 1-fold up and down, respectively, and the horizontal line represents a *p*-value of 0.05. **b** Heatmap plot of differentially expressed genes in human endometrial cancer. **c** GO and KEGG pathway analysis of intersection genes (Top 10 significance). **d** The expression of real-time qPCR was in accordance with microarray data. (*p < 0.05, **p < 0.005)

We performed a microarray analysis, comparing the global mRNA expression profile between HEC-1-B and CypB-knockdown HEC-1-B cells. A volcano plot of the identified quality-controlled genes (*p* < 0.05; fold change, > 1) is presented in Fig. 3a. The microarray identified 1536 differentially expressed mRNAs in total, of which 652 were upregulated and 884 downregulated in the CypB-knockdown group. A heat map was generated to show genes that were previously identified as significantly upregulated in HEC-1-B cells (Fig. 3b).

### GO functional and pathway enrichment analysis

GO and pathway enrichment analysis showed that the DEGs were significantly enriched in the cell cycle, glycosphingolipid biosynthesis, adherens junctions, and metabolism pathways. Important genes and pathways involved in this process are shown in Fig. 3c.

### Validation of differentially expressed RNA by qRT-PCR

To evaluate the reliability of microarray data, we verified the expression of five differentially expressed mRNA in HEC-1-B cells by qRT-PCR assay. As shown in Fig. 3d, the mRNA expression of SREBF1, SPP1, PPIB, and PLAT was significant downregulation in the CypB-knockdown cells. While the CDKN1A mRNA shown a much higher expression in the CypB-knockdown cells. All these results were consistent with the microarray data.

## Discussion

Endometrial carcinoma remains one of the leading causes of death among women, it is crucial to novel molecular targets for its diagnosis, prognosis, and treatment for improving the clinical strategy and outcome for this disease. Cyclophilins have been implicated in a variety of cancers; however, their expression has not been studied in endometrial carcinoma. In the present study, we screened the CypB expression pattern in endometrial cancer and found that CypB was an oncogene, which was upregulated in endometrial cancer, and downregulation of CypB inhibits HEC-1-B cell proliferation and metastasis.

Recent studies have found that CYPA expression is implicated in several cancers, including lung cancer [27, 28], pancreatic cancer [29, 30], hepatocellular cancer [31], and buccal squamous cell carcinoma [32], and that it might play a role in apoptosis through the activation of caspases and apoptosis-inducing factor. A study with two-dimensional gel electrophoresis and MALDI-Q-TOF MS/MS-based proteomics approach found that overexpression of CYPA is significantly correlated with a low degree of cancer differentiation, and its overexpression was associated with decreased survival in endometrial carcinoma [9].

On the other hand, the structurally similar CypB, which was found in the endoplasmic reticulum, has been implicated in STAT3 activation and the generation of reactive oxygen species in cancer cells [24]. CypB facilitates the transcriptional activity of STAT5 by inducing the release of the repressor PIAS3, resulting in significantly enhanced STAT5-mediated gene expression [14, 18]. At the cell surface, CypB also serves as a ligand for the CD147 receptor [33], which regulates MAPK activation, motility, calcium transport [33–35], and the expression of the pro-apoptotic protein BIM [36]. Gene expression studies revealed that CypB is highly upregulated in malignancies. Ablation of CypB expression in glioblastoma multiforme cells suppresses several canonical oncogenic signaling pathways, including mutant P53, MYC, and CHK1. Teng and colleagues revealed that CypB was overexpressed in NSCLC and inhibition of CypB could suppress cell proliferation, migration, invasion, and angiogenesis via regulating the STAT3 pathway [37]. Notably, angiogenesis plays an important role in tumor progression. In this study, we detected the expression of VEGF and Ang2, which are two important angiogenesis-related proteins, and we found that VEGF expression was starkly been inhibited with the downregulation of CypB, while not for Ang2 (Figure S1). CM Holland and colleagues found that VEGF-B mRNA was significantly lower in endometrial cancer than benign endometrium, while the expression level of Ang2 mRNA in endometrial carcinoma was higher than that in benign endometrium, but there was no statistical significance. The results of this study are consistent with ours at the mRNA level [38].

## Conclusions

In this study, we demonstrated endometrial tumor tissue exhibited a significantly higher expression of CypB, suggesting that CypB expression could be considered an effective indicator for the clinical outcome of endometrial cancer. Furthermore, our results demonstrate that CypB acts as an oncogene in endometrial cancer.

## Supplementary Information

**Additional file 1.**

**Additional file 2.**

## Data Availability

In consideration of patient privacy, the datasets involved in this study are not publicly available, but the datasets generated and/or analyzed during the current study are available from the corresponding author on reasonable request.

## Abbreviations

CypA: Cyclophilins A
CypB: Cyclophilins B
shRNA: Short hairpin RNA
NC: Negative control
CCK8: Cell Counting Kit-8
qRT-PCR: Quantitative real-time PCR
SDS-PAGE: Sodium dodecyl sulfate-polyacrylamide gel electrophoresis
PVDF: Polyvinylidene fluoride
IHC: Immunohistochemistry
TMA: Terminal Market Association
EDTA: Ethylenediamine tetraacetic acid
GO: Gene ontology
KEGG: Kyoto Encyclopedia of Genes and Genomes
FDR: False discovery rate
STAT5: Signal transducer and activator of transcription 5
PIAS3: Protein inhibitor of activated STAT3
CHK1: Cell cycle checkpoint kinase 1
VEGF: Vascular endothelial growth factor
Ang2: Angiopoietin 2
