# Supplementary material for: CypB promotes cell proliferation and metastasis in endometrial carcinoma
Source: BMC Cancer. 2021 Jun 29;21:747. doi: 10.1186/s12885-021-08374-7 (PMC8240271; doi:10.1186/s12885-021-08374-7)
Supplement: Supplementary file 1 — Additional file 1. [file 12885_2021_8374_MOESM1_ESM.pdf]

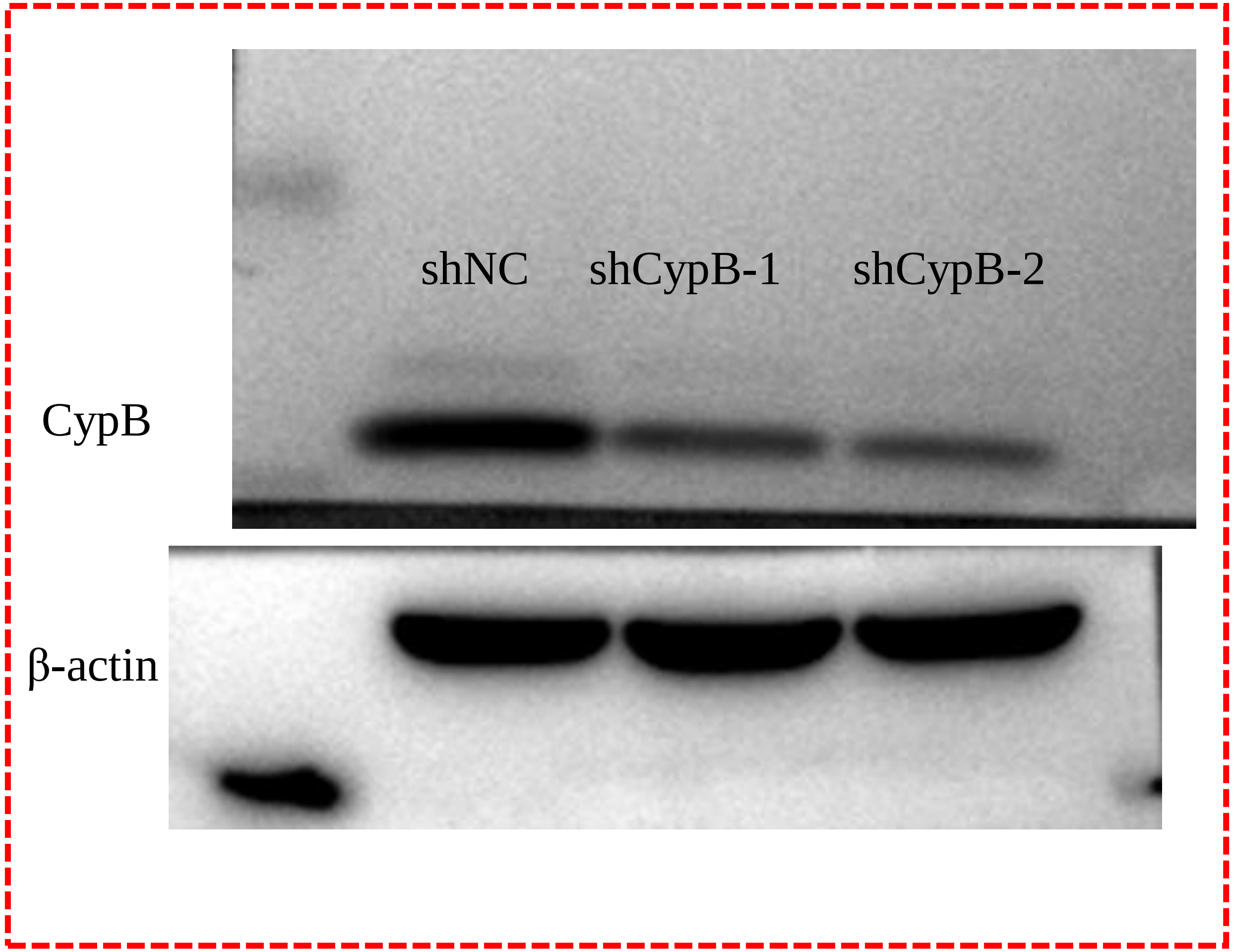

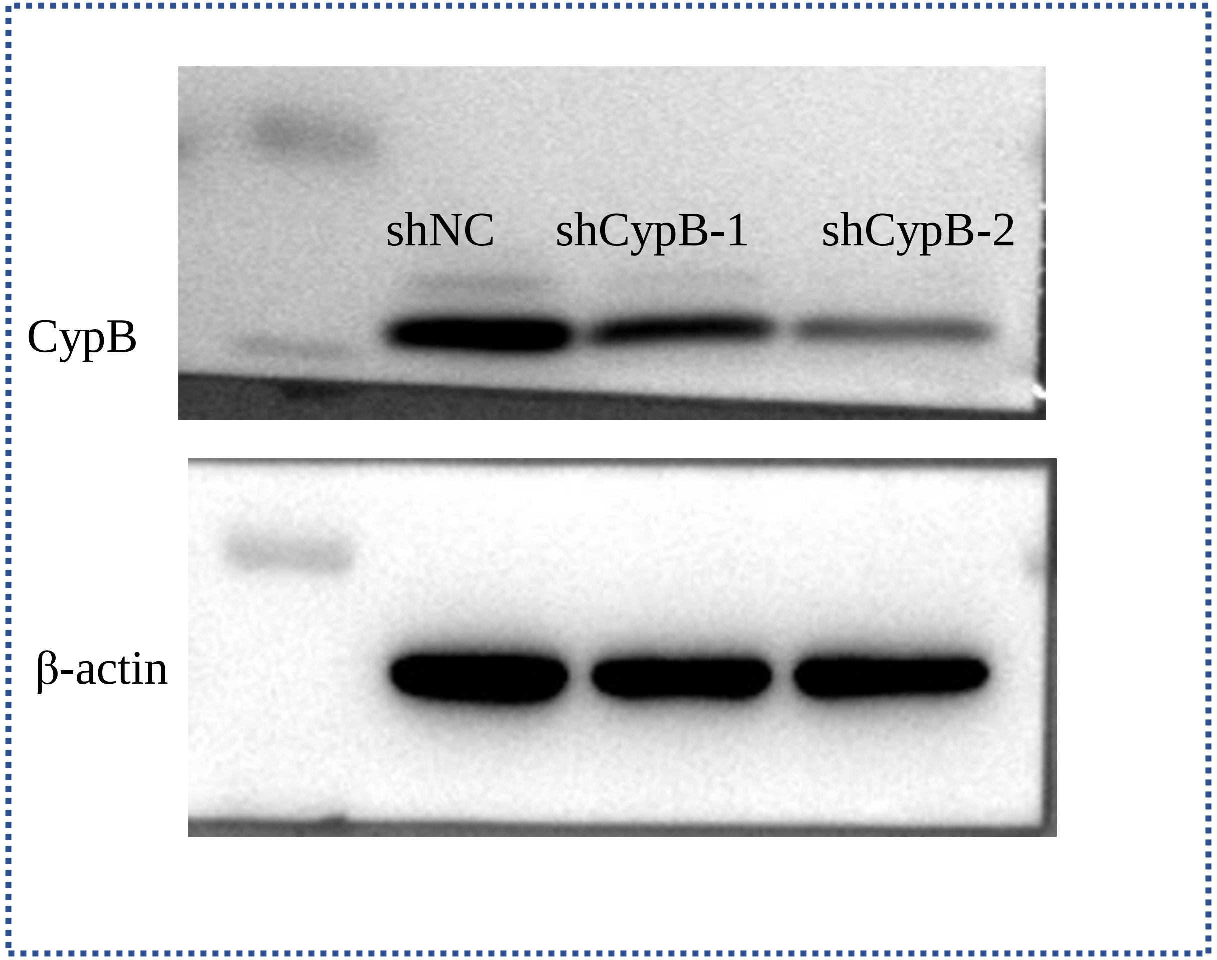

Figure S1. shCYPB-1 and shCYPB-2 significantly downregulated the expression of CypB in protein level. The conventional microscope pictures of HEC-1-B cells treated with shNC or shRNAs (-1 and -2).

(Two representative WB results were obtained, the results shown in the manuscript were Cropped from image marked with red dotted box.)

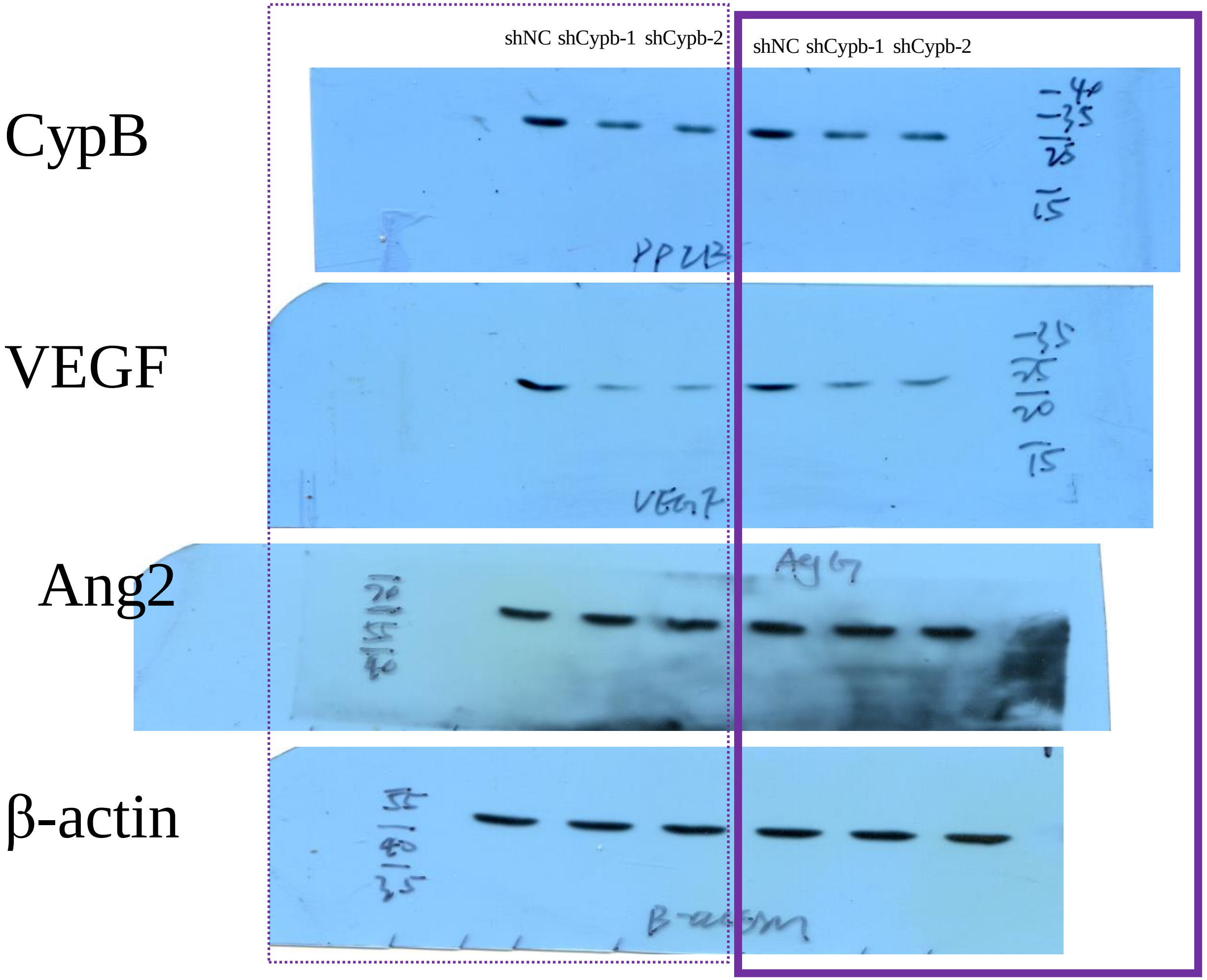

Figure S2. The protein expression of VEGF and Ang2 after CypB-downregulation in HEC-1-B cells. (The representative WB results were obtained, the results shown in the manuscript were cropped from image marked with purple solid wire frame.)
