# Supplementary material for: CypB promotes cell proliferation and metastasis in endometrial carcinoma
Source: BMC Cancer. 2021 Jun 29;21:747. doi: 10.1186/s12885-021-08374-7 (PMC8240271; doi:10.1186/s12885-021-08374-7)
Supplement: Supplementary file 2 — Additional file 2. [file 12885_2021_8374_MOESM2_ESM.pdf]

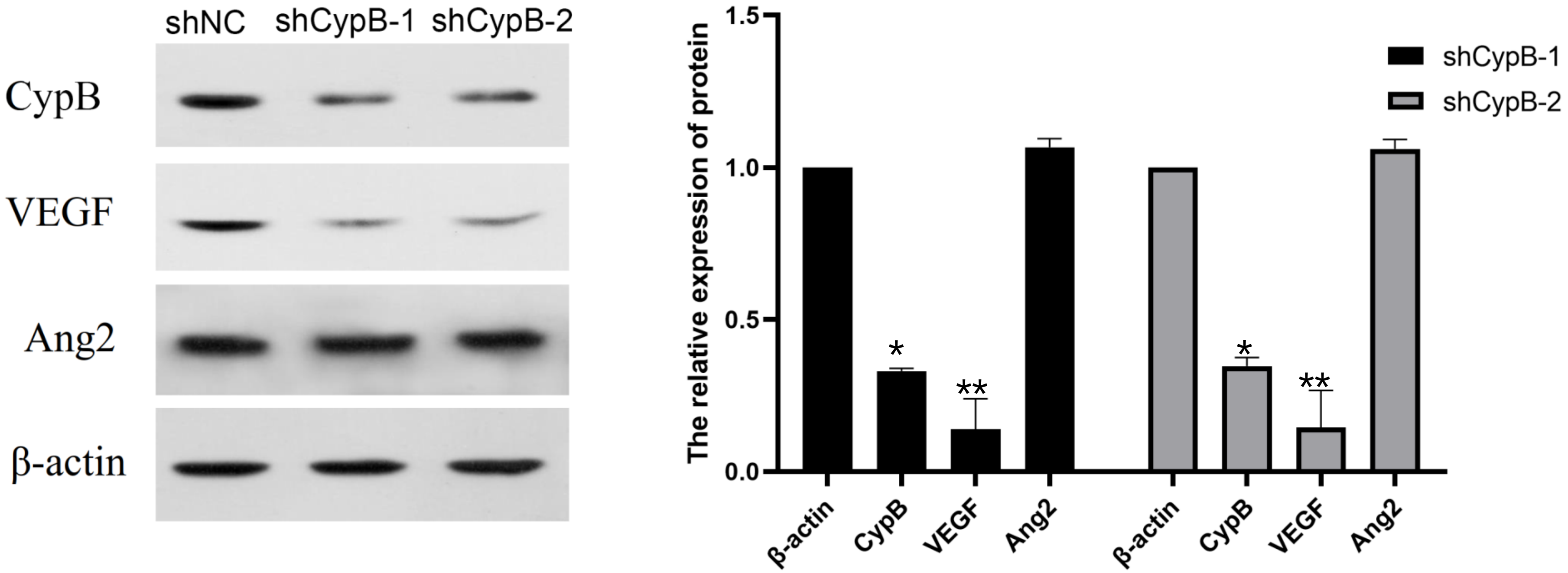

Figure S1. The protein expression of VEGF and Ang2 after CypB-downregulation in HEC-1-B cells. The representative cropping bands are present. The relative expression levels were determined with ImageJ software. β-actin was used as a loading control. The data shown are from three independent experiments. (*$p < 0.05$, **$p < 0.005$).
